# Plant‐based production can result in covalent cross‐linking of proteins

**DOI:** 10.1111/pbi.13598

**Published:** 2021-05-04

**Authors:** Roger Castells‐Graells, George P. Lomonossoff

**Affiliations:** ^1^ Department of Biological Chemistry John Innes Centre Norwich Research Park Colney UK; ^2^ Department of Chemistry and Biochemistry University of California Los Angeles CA USA

**Keywords:** molecular farming, virus‐like particle, *Nicotiana be* *nthamiana*, insect cells, recombinant protein, transient expression, protein interactions

Antibodies, antigens and enzymes for replacement therapies and virus‐like particles (VLPs) have all been produced successfully in plants as part of the concept of ‘Molecular Farming’ (Lomonossoff and D’Aoust, 2016). There have been several differences noted between plant‐expressed proteins, and their equivalents produced in other systems, such as CHO cells, particularly regarding their glycosylation. However, several publications have also indicated that preparations of plant‐expressed proteins, including antibodies, VLPs and soluble molecules such as HIV gp120, have a higher proportion of multimers or aggregates than their CHO‐ or yeast‐expressed equivalents (e.g. Mechtcheriakova *et al*., 2006; Ramessar *et al*., 2008; Rosenberg *et al*., 2013). The cause of this has not been investigated in detail because the molecules expressed in the different systems are often not identical and may have been purified to different extents.

As part of our studies on virus maturation, we have expressed the coat protein of the insect virus, *Nudaurelia capensis* omega virus (NωV) in both *Nicotiana benthamiana* (a heterologous expression system) and insect cells (the homologous system). NωV belongs to the *Tetraviridae*, a family of viruses with non‐enveloped T = 4 capsids and single‐stranded positive‐sense RNA genomes that infect Lepidoptera. Maturation of the virus particles from procapsid to mature capsid involves a compaction of the particles from 48 to 41 nm diameter with a concomitant autocatalytic cleavage of the full‐length coat protein (α‐peptide) to the β and γ peptides, with sizes of 70, 62 and 8 KDa, respectively. Experiments with NωV coat protein expressed in insect cells have shown that this maturation can be triggered by reducing the pH of a suspension of procapsids from 7.6 to 5.0 *in vitro* (Canady *et al*., 2000). We have recently shown that mature particles extracted from *N*. *benthamiana* leaves expressing the α‐peptide are very similar in structure from the mature particles derived from insect cells and to contain the cleaved β and γ peptides (Berardi *et al*., 2020; Castells‐Graells, 2019). This indicates that the maturation process can be faithfully recapitulated in plants. By contrast, when particles were extracted from leaves as procapsids at pH 7.6 and then matured *in vitro* by reducing the pH to 5.0, the kinetics of cleavage of the α‐peptide were consistently slower and the process less complete than found with the equivalent material derived from insect cells (Castells‐Graells, 2019).

To investigate potential differences between plant and insect cell‐derived procapsids, genes encoding the identical amino acid sequence of the α‐peptide were expressed in *N*. *benthamiana* leaves and insect cells using plasmids pEAQ‐*HT*‐NωV and pFastBac‐NωV, respectively (Agrawal and Johnson, 1995; Berardi *et al*., 2020; Castells‐Graells, 2019); in both cases, VLPs were extracted in the procapsid form as previously described. For the plant‐derived sample, procapsids were separated from mature capsids by sucrose gradient centrifugation, since some maturation occurs within the cells over time. While the procapsids isolated from insect cells contained almost exclusively the monomeric α‐peptide, the plant‐derived material contained additional higher molecular mass bands which appear to be multimers of the protein (Figure 1a). This is consistent with the previous identification of dimers by mass spectrometry and Western blotting in samples of plant‐expressed NωV VLPs (Castells‐Graells, 2019). Since identical conditions were used to extract and purify the procapsids and the denaturing conditions used for the SDS‐PAGE analysis were the same in each case, the formation of the oligomers must be a specific consequence of using plants for expression. Given the fact that the oligomers resisted denaturation, it is probable that they result from covalent cross‐linking of subunits.

**Figure 1:**
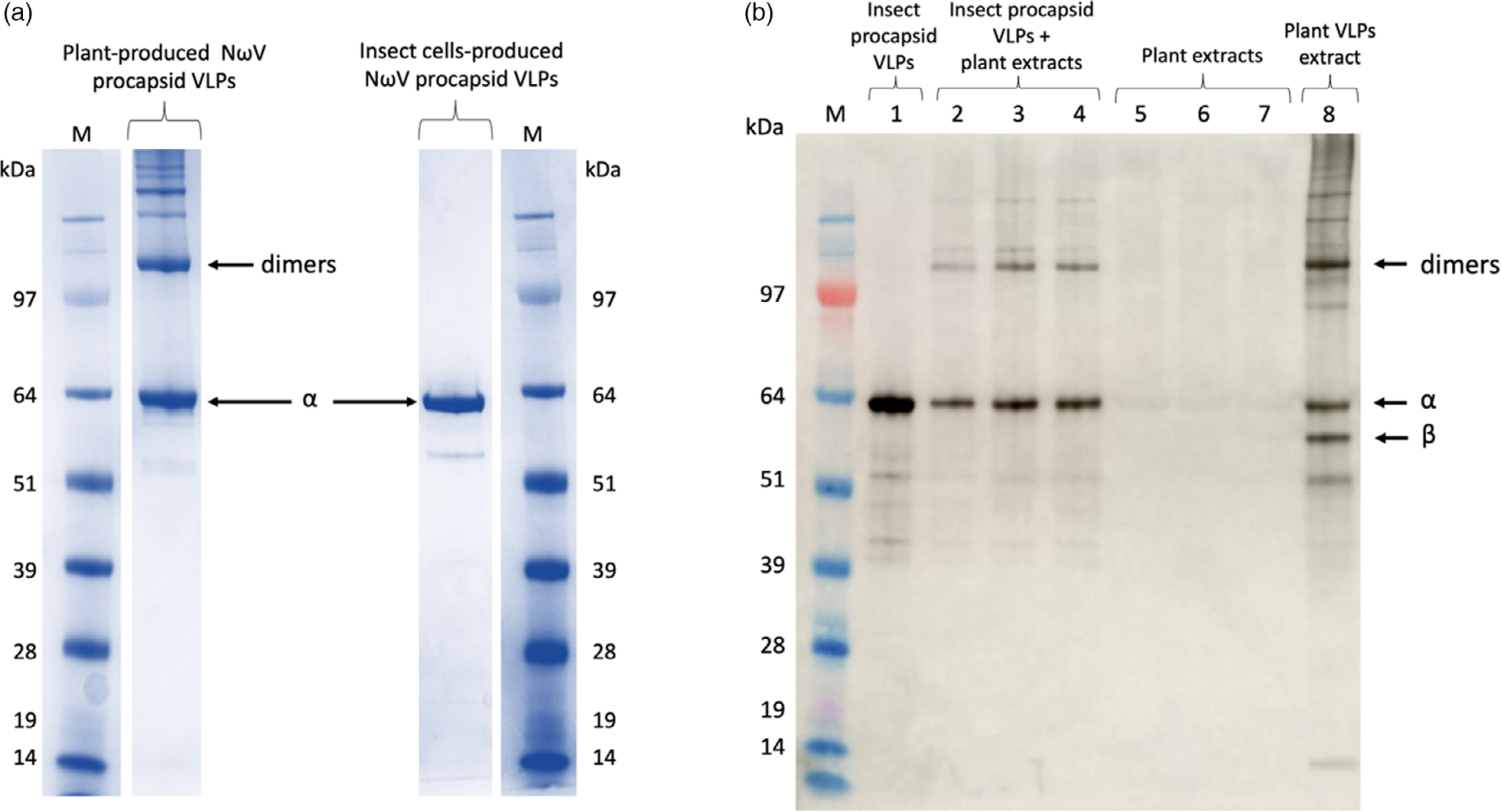
Side‐by‐side comparison of NωV WT VLPs produced in plants and insect cells and the effect of incubation of insect cell‐produced NωV WT VLPs with plant extracts. (a) Comparison of plant‐ and insect cell‐produced NωV procapsid VLPs. Samples were denatured in LDS loading buffer containing β‐mercaptoethanol. Following electrophoresis on 4‐12% (w/v) Bis‐Tris NuPAGE gels, the proteins were stained with InstantBlue (Expedeon). The positions of the α‐peptide and dimers are indicated. Additional high‐molecular mass bands are present only in the plant‐produced sample. (b) Western blot of insect cell‐produced NωV VLPs incubated with extracts from differently treated plants. In lanes 1 to 4, NωV VLPs produced in insect cells were mixed with pH 7.6 buffer (1) or with different plant extracts (2, 3 and 4). Lanes 5 to 7 represent the corresponding plant extracts used in lanes 2 to 4 but without the added VLPs. Lane 8 contains an extract from plants infiltrated with pEAQ‐HT‐NωV‐WT. The plant extract in lanes 2 and 5 was from uninfiltrated plant leaves, in lanes 3 and 6, from plants infiltrated with pEAQ‐HT‐EV and for lanes 4 and 7, from plants infiltrated with pEAQ‐HT‐GFP. In all cases, the plant material was blended with the pH 7.6 buffer and the immunodetection was with a polyclonal antibody for the NωV coat protein. M = SeeBlue Plus 2 pre‐stained protein standards.

To investigate whether cross‐linking occurred within the plant cells or during extraction, we incubated insect cell‐produced NωV procapsids with plant extracts prepared in the same buffer used to extract procapsids (50 mm Tris‐HCl pH 7.6, 250 mm NaCl) and in which cleavage of the α‐peptide does not occur. The conditions of the incubation (4 h at 4 °C) were approximately the same as those used to prepare procapsids up to the sucrose gradient step. Western blot analysis (Figure 1b) showed that incubation of insect cell‐produced procapsids in plant extracts results in the appearance of dimers and additional high molecular bands (lanes 2 to 4), not seen when the procapsids were incubated in buffer alone (lane 1). This pattern of higher bands is similar to that found in plant tissue expressing the α‐peptide (lane 8), though in this sample maturation products are also seen since the procapsids were not purified. The formation of dimers occurred irrespective of whether the insect cell procapsids were incubated with extracts prepared from uninfiltrated leaves (lane 2), leaves infiltrated with the empty pEAQ‐*HT* vector (pEAQ‐*HT*‐EV; lane 3) or leaves incubated with pEAQ‐*HT*‐GFP (lane 4), indicating that the act of infiltration or the presence of Agrobacterium did not influence the result. The negative controls (lanes 5 to 7) containing the equivalent plant extracts without the added procapsids did not show any NωV‐specific bands demonstrating the specificity of the antiserum. These results strongly imply that some component present in the plant extracts, such as peroxidases, induces the cross‐linking of the NωV coat proteins and that this cross‐linking occurs during extraction and purification. Varying the conditions of extraction using, for example, buffers at different pHs (5.0, 7.6, 10.0) or adding DTT to 1mM did not make a detectable difference to the result.

The observation of cross‐linking has implications for the production of proteins in plants. In the case of NωV, we propose that such cross‐linking is detrimental to the protein rearrangements necessary for the efficient *in vitro* maturation of the procapsids, thus affecting the final assembly. We conclude that it is the time taken for the isolation and purification of the procapsids (approximately 4 h) that allows the cross‐linking to occur, thereby interfering with the maturation process *in vitro*; however, cross‐linking within cells prior to extraction might also occur. Cross‐linking can still occur post‐maturation, but this does not affect the structure of the particles.

Though cross‐linking is clearly deleterious for the maturation of NωV capsids, in certain cases it may not be harmful or even beneficial. For example, in the case of plant‐produced antibodies, the presence of aggregates increased the IC_50_ of preparations several fold compared to CHO‐produced material (Ramessar *et al*., 2008; Rosenberg *et al*., 2013). Likewise, partial cross‐linking of subunits can also aid the stability and structural integrity of VLPs (Peyret *et al*., 2015); indeed, VLPs and virus particles intended for vaccine purposes are often deliberately cross‐linked during formulation. Nonetheless, it is important to be aware of cross‐linking in plant‐produced material so that methods for its control or elimination can be developed. If the enzymes involved can be identified, cross‐linking could be addressed by removing them, using genetic engineering techniques like CRISPR‐Cas9 (Belhaj *et al*., 2015). Controlling cross‐linking should increase interest using plants for the production of pharmaceuticals and other relevant biological products (Lomonossoff and D’Aoust, 2016).

## Conflict of interest

G.P.L. declares that he is a named inventor on granted patent WO 29087391 A1 which describes the HyperTrans expression system and associated pEAQ vectors used in this manuscript.

## Author contributions

R.C‐G and G.P.L conceived the study. R.C‐G. designed the constructs and conducted expression tests in plants and insect cells. G.P.L. supervised the work and provided scientific advice. Both authors contributed to writing and editing the manuscript.
